# 

**Published:** 1883-03-20

**Authors:** 


### *EDITORIAL NOTICES.*

UNITED STATES DISPENSATORY.—See announcement of the last edition of the United States Dispensatory in this Journal.

PROF. THAD. JOHNSON, who has been long confined to his room with a severe nervous affection, is, at this writing, reported better, and his friends are hopeful of his recovery.

LACTO-PHOSPHATES.—Examine the new advertisement in this issue of a preparation prepared by Dr. Wheeler, of Montreal, Canada—*Elix. Ferri et Calcis Phosphate.*

ANATOMY IN THE SOUTHERN MEDICAL COLLEGE.—The facilities for the study of Anatomy in *The Southern Medical College*, Atlanta, are of a superior order—cannot, indeed, be surpassed in any Institution.

LISTERINE.—See new advertisement of Lambert & Co., Manufacturing Chemists, St. Louis, in this Journal. Listerine, as prepared by this house, is having a fine run, and is coming into general use as an antiseptic preparation in surgical and obstetrical practice, etc.

DEATH OF A. H. STEPHENS, GOVERNOR OF GEORGIA.—At a late meeting of the Board of Trustees of the Southern Medical College, Atlanta, very strong resolutions were adopted as a tribute to MR. STEPHENS, who was a member of the Board and a warm friend of the Institution. He was truly a great friend and patron of Education, himself a profound scholar—a wise philosopher, a great statesman; and in all respects, a great and good man.

MEDICAL ASSOCIATION OF GEORGIA.—Remember, the State Medical Association meets the present year on the third Wednesday of April, at Athens, Georgia.

Dr. R. M. WADE, Secretary of the Committee of Arrangements, at Athens, writes: "That the court-house has been selected for the place of meeting of the Georgia Medical Association. Board at the two hotels reduced to two dollars per day.

"The programme of business and entertainment will be issued as soon as the arrangements have been perfected. We are working to make the visit of the Association pleasant as well as profitable."

*THE LOUISIANA STATE MEDICAL SOCIETY,*

Which held its last meeting in New Orleans, March 30th, 1881, and which adjourned to meet at the same place in March, 1882, but which failed to make the last appointment by reason of devastating floods, has by consultation of the officers, been appointed to meet in the city of Shreveport, on Wednesday, the 4th day of April, 1883.

The same officers and committee- last elected hold their positions under the constitution. The above item of information was kindly furnished us by A A. Lyon, M. D. President.

### *RECEIPTED.*

1883.—Drs. J I Grover, S W Eaton, W T Kendall, M W Speer, W P Walker, J C Smith, J P Allread, J W Albert, E Van Goidtsnoven, J L Richards, D R Norman, H W Kennerly, D B Searcy, Jno H Young, T J Jones, J H Boggan, W E Quinn, K H Davis, N B Warren, D E Ruff, J M Boring, R Fox, J F Davis, E H M Parrham, A C Fox, to Sept. '83; C P Sanders, C B Thomas, C M Gibson, I N Goss, to July, '83; A G Grove, to July, '83; G Wright, to July, '83; A R Jones, to July, '83; J M Boring, 1882; J L Carothers, J P Stevens H L Sutherland, J B Bailey, J W Sanders, 1882; R W Rea, 1882.

